# Supplementary figures and images for: Positive correlation between transcriptomic stemness and PI3K/AKT/mTOR signaling scores in breast cancer, and a counterintuitive relationship with PIK3CA genotype
Source: PLoS Genet. 2021 Nov 11;17(11):e1009876. doi: 10.1371/journal.pgen.1009876 (PMC8584750; doi:10.1371/journal.pgen.1009876)

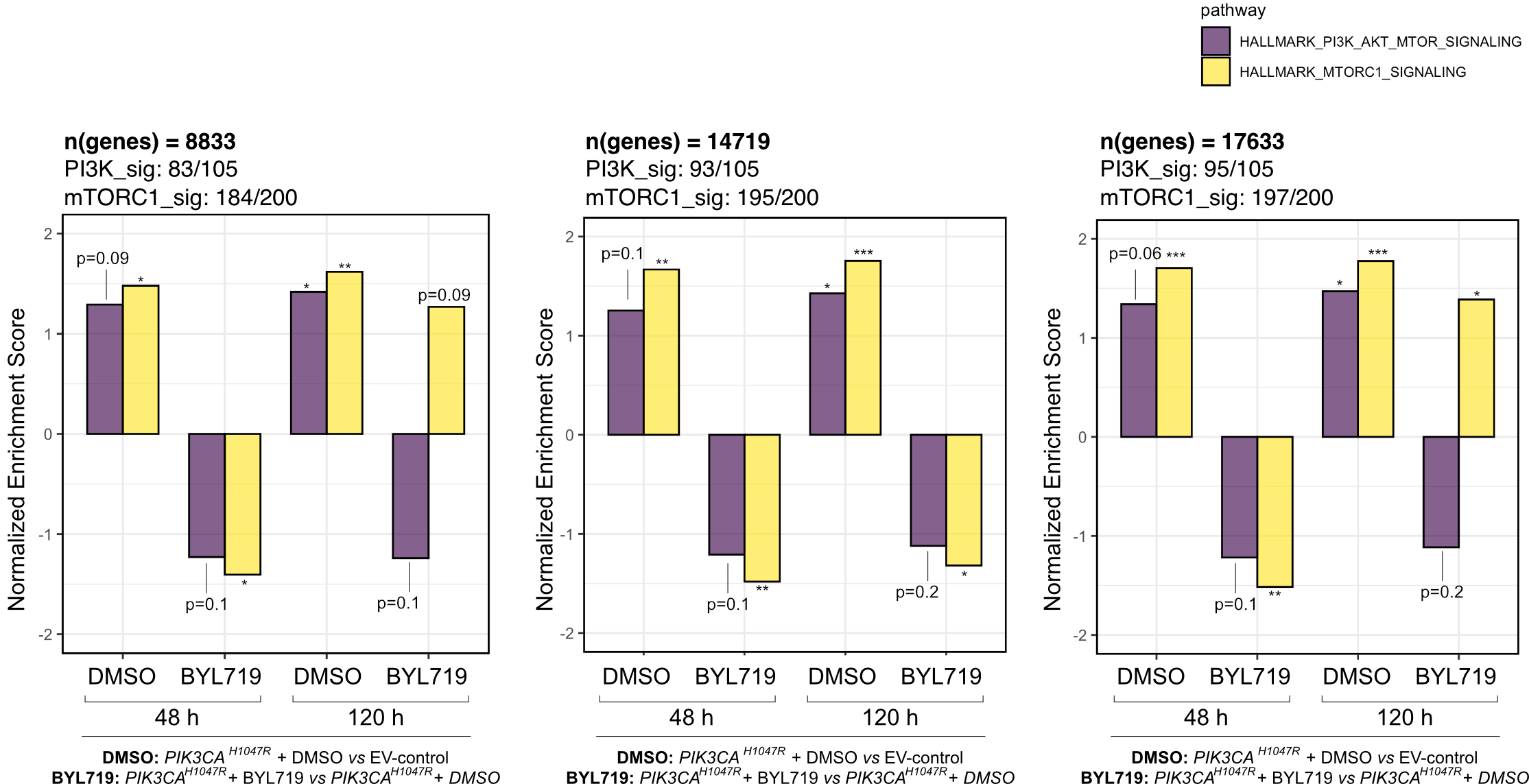

Supplement: S1 Fig — Each plot corresponds to replicate analyses of the MCF10A transcriptomic data in Fig 2 (main manuscript), following different filtering thresholds for absolute gene expression. The total number of ranked genes and their overlap with the tested signatures are shown above each analysis. The p-values correspond to each enrichment’s significance following 100,000 permutations of the gene ranks; * p ≤ 0.05, ** p ≤ 0.01, *** p ≤ 0.001; FDR = 0.05. (TIFF) [file pgen.1009876.s001.tiff]

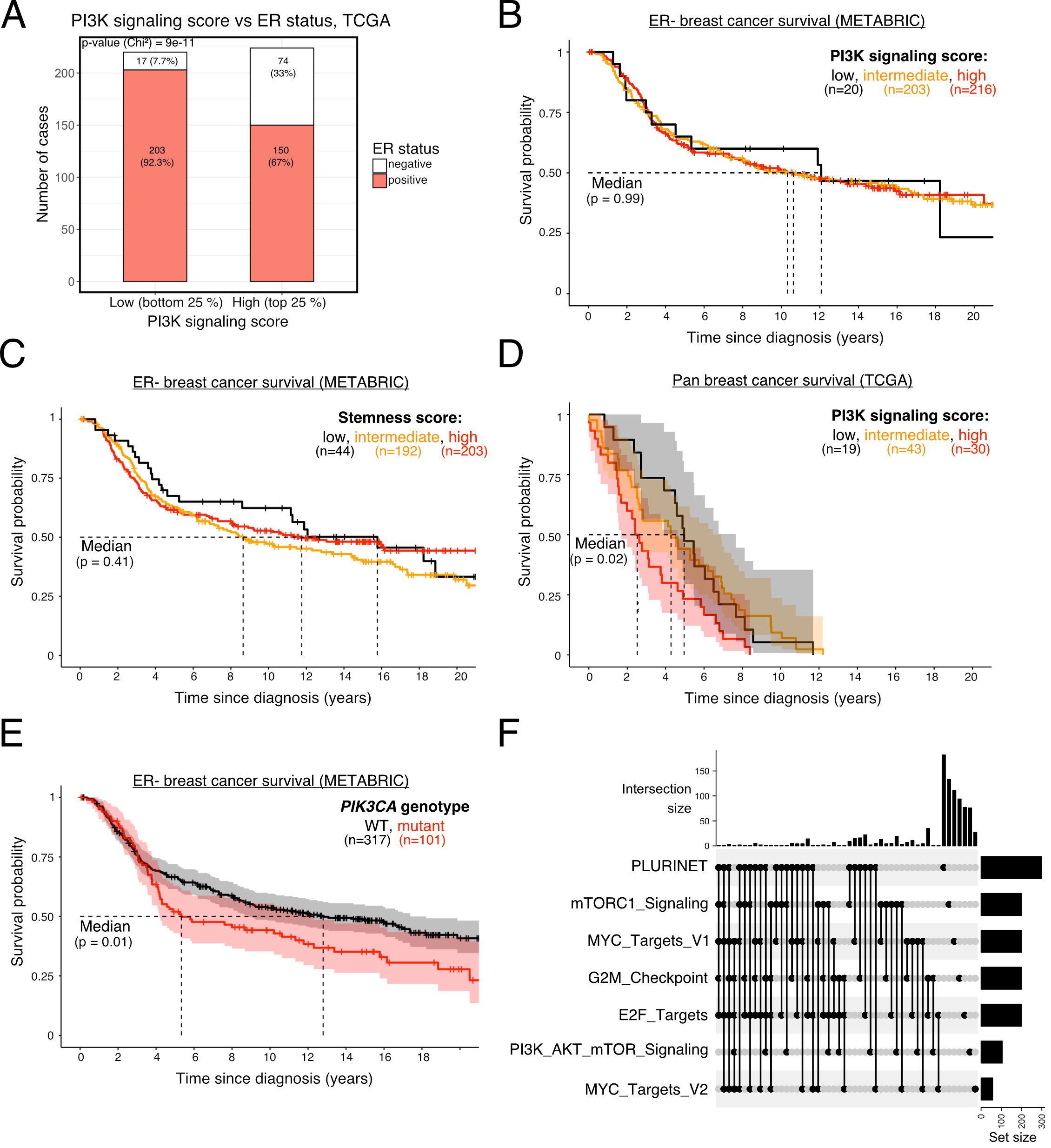

Supplement: S2 Fig — (A) PI3K signaling score distribution in TCGA breast tumors stratified according to ER status. Survival analysis in estrogen receptor (ER)-negative breast cancer patients, as a function of PI3K signaling (B) or stemness (C) score. (D) Pan-breast cancer patient survival in TCGA, as a function of PI3K activity score. (E) ER-negative breast cancer patient (METABRIC) survival as a function of binary PIK3CA genotype. The sample size for each panel and subgroup is indicated, and p-values were calculated using a log-rank test; where shown, the 95% confidence intervals are indicated by shading. (F) UpSet plot showing intersection set sizes across the specified gene set combinations. (TIFF) [file pgen.1009876.s002.tiff]
