# Supplementary material for: Positive correlation between transcriptomic stemness and PI3K/AKT/mTOR signaling scores in breast cancer, and a counterintuitive relationship with PIK3CA genotype
Source: PLoS Genet. 2021 Nov 11;17(11):e1009876. doi: 10.1371/journal.pgen.1009876 (PMC8584750; doi:10.1371/journal.pgen.1009876)
Supplement: Attachment — Submitted filename: 2021-06_Rebuttal_PI3K_stemness_comp_MS_RRM_FINAL.docx [file pgen.1009876.s005.docx]

------------------------------------------------------------------------------

Attn	Sara Monaco, PhD

Managing Editor

Review Commons

Dear Dr. Monaco,

We are grateful for the Reviewers’ time assessing our manuscript and address each of their comments in turn below. Revisions to the submitted work are highlighted, alongside exact references to the main text. We have also allowed ourselves a minor revision to the title to better reflect the main findings of our study.

As further detailed in the rebuttal to the Referees, we hope that our report may spur evaluation of the potential clinical benefits of integrating conventional genetic PI3K pathway information with transcriptomic indices of its functional activation in breast cancer. This could be particularly relevant for aggressive breast cancer types with low prevalence of *PIK3CA* mutations, which are nevertheless characterised by both high stemness and PI3K signalling scores. These findings may therefore prompt further investigation of combining PI3K inhibitors with pro-differentiation therapies in this setting.

Sincerely yours,

Ralitsa R. Madsen

(*on behalf of all authors)*

**Reviewer #1**

*Evidence, reproducibility and clarity:*

The manuscript from Madsen et al. describes some bioinformatics investigations into relationships between PI3K, stemness, and survival in breast cancers. There are claims that multiple PI3K mutations (referred to as dosage) are needed to induce stemness, and that stemness is related to PI3K activity score, a transcriptomic readout of PI3K pathway, and both could stratify breast cancer survival. Overall, I found the evidence for these claims to be underdeveloped and largely correlational. In Figure 1, correlation is shown between PI3K activity score and stemness, but how does that correlation look for other "scores"? The authors could stratify patient outcomes somewhat by PI3K activity score (Fig. 2), but how significant is that compared to other scores? Does it prove to be causal, that is, inform drug choice? With regards to "dosage" claims, there were no phenotypic markers of PI3K pathway activity and stemness measured in response to targeted perturbations of either. Thus, I believe the publication of this manuscript to be premature.

*Significance:* 

Without addressing fundamentals in the above there is no use for this.

**AUTHORS’ RESPONSE:**

*We note that the other two reviewers were positive about our manuscript, and while we understand the important points about empirical validation of our findings both in clinical trials and with reference to tissue analysis raised by the reviewer, we believe he/she is raising the bar unreasonably high for this report. Our findings and their interpretation have important translational implications that warrant further study, and bringing these to wider attention at this stage we believe is warranted. Indeed, we would emphasise that the presented work is similar in scope (albeit narrower in focus) to high-profile computation-only publications, which have proven impactful over time (particularly in hypothesis generation and method application). Examples include but are not limited to Sanchez-Vega et al. Cell 2018 (Oncogenic Signaling Pathways in The Cancer Genome Atlas; 954 citations)* [1] *and Malta et al. Cell 2018 (Machine Learning Identifies Stemness Features Associated with Oncogenic Dedifferentiation; 420 citations)* [2]*. The other reviewers accept the scope of our study.*

*When it comes to the specificity of the PI3K and stemness scores, we emphasise that we perform multiple independent checks to demonstrate that each score associates with previously published phenotypes. We demonstrate consistent results with different PI3K and stemness scores which adds strengths to our study. Additional details are provided in our response to Reviewer 2. Moreover, this study is not simply a fishing experiment devoid of a defined prior hypothesis – what we are doing is following up on strong, prior mechanistic findings in cellular models which we previously established.*

*While targeted perturbations are difficult to analyse, given the need to access patient data, we consider the current analyses worthy of publication for the benefit of the community. Patient stratification has been a huge challenge in this field, and even though we are not in a position to take this forward with clinical studies, it is clear that others may be able to, and this is also what is proposed in our manuscript.*

**Reviewer #2**

*Significance:* 

These interesting observations highlight the limitations of single gene mutation as biomarker in cancer. Indeed simple binary classification of tumours into PIK3CA WT vs. mutant, without considerations for allele dosage and pathway activation status can be misleading.

**AUTHORS’ RESPONSE:**

*We thank the Referee for these positive comments.*

*Evidence, reproducibility and clarity:*

Experimental evidence in preclinical models has demonstrated a link between PI3K pathway oncogenic hyperactivation and dedifferentiation, reactivation of multipotency, and lineage disruption. However, the correlation between PI3K pathway hyperactivation and stemness in human remained elusive. In the present study, Madsen et al. addressed whether PI3K signaling and stemness signatures correlate in both METABRIC and TCGA cohorts.

The manuscript is well written, the analyses are thorough, and data well presented. This study reports several interesting observations. The PI3K activity score is highest in the TNBC subgroup which is the one that encompasses the least number of PIK3CA mutated tumors. These observations emphasize and reflect the fact that the PI3K pathway can be activated by means other than *PIK3CA* mutation (PTEN loss, RTK pathway activation...). In ER+ tumors, the mutation status of the PIK3CA gene does not predict OS in the METABRIC cohort but the PI3K activity score does. In ER- tumors, the mutation status of the PIK3CA gene predict OS in the METABRIC cohort but not the PI3K activity score. There is no correlation between PI3K activity score and the PIK3CA mutation status in both TCGA and METABRIC cohorts. The authors also report that heterozygous mutation of PIK3CA decreases the PI3K activity score as compared to WT. And that homozygous mutation of PIK3CA does not increase the PI3K activity score as compared to WT.

Some of these observations would require in-depth functional validation, however this is beyond the scope of this study. Several points need to be addressed before envisioning publication.

**Major points:**

1) The authors should use alternate PI3K signaling gene signature (2 or 3) available in the different resources or within the C2 curated dataset to confirm the correlation with Stemness in the METABRIC cohort (As they did for the Stemness signature from Malta and Miranda et al,. against the HALLMARK_PI3K_AKT_MTOR signature). This would strengthen the whole study as the article relies on the sole use of the HALLMARK_PI3K_AKT_MTOR gene signature from GSEA, with the postulate that it is faithfully reflective of PI3K activity in breast cancer (which is partially true given the tissue specificity of this pathway).

**AUTHORS’ RESPONSE:**

*We agree that the use of isolated signatures is insufficient for this type of work. However the mSigDb Hallmark PI3K signature that we have used is an ‘amalgamation’ or ‘metanalysis’ of all other PI3K signatures submitted to mSigDb. We reasoned that a consensus signature that combines information from multiple gene sets available within the mSigDb database, including C2 signatures (Liberzon et al. 2015 Cell Systems)* [3]*, would be superior to isolated signatures which may be biased by study-specific factors. Moreover, the ease with which our approach can be extended to other pathways, alongside the annotated code provided, should make it easy to implement and build upon by other researchers. We had, however, already included analysis using an independent score based on the PROGENy (Perturbation-RespOnse GENes) methodology (Schubert et al. 2018 Nature Communications* [4]*), which is fully independent from the method used to generate the mSigDb hallmark signatures yet also relies on transcriptional “footprints” to build a signature of pathway activation (see Fig.1D/1E and lines 162-167).*

*Nevertheless, in response to the Referee’s comment, we have now performed additional analyses using two independent PI3K signatures from Jin et al. 2020 Nature* [5] *(one derived on the basis of mutant PIK3CA overexpression in breast epithelial cells, and the other on the basis of PI3K inhibitor treatment in cMAP) – interestingly, these exhibit an even stronger correlation with stemness indices, in addition to a positive correlation with the alternative PI3K signature scores based on PROGENy and the mSigDb’s Hallmark PI3K signatures. These results are included in the revised Fig.1D and Fig.1E.*

*It is further worth pointing out that the utility of our score is supported by our ability to reproduce major findings from prior studies, including:*

1. *A negative correlation between PI3K activity score and PIK3CA mutation status when mutation dose* ***is not*** *taken into account (one of our main points, of course, is that this must be considered moving forward). Additional functional support for this pan-cancer is provided in Figure 5A of Zhang et al.’s 2017 Cancer Cell* [6]*, where knockdown of PIK3CA correlates with increased PI3K pathway activity, in contrast to the expected decrease in pathway activity when AKT and mTOR components are knocked down.*
2. *A quantitative relationship between the strength of the PI3K pathway score and overall patient survival (breast cancer in our study, but also shown to apply pan-cancer in Zhang et al.* [6]*).*
3. *Highest PI3K pathway activity score in TNBC and HER2+ samples, followed by Luminal B tumours (see Fig.3 in Koboldt et al. 2012 Nature* [7]*; Extended Fig. 6E in Jin et al. 2020 Nature* [5]*) – as we demonstrate, a similar pattern is also observed for the stemness score. It is worth pointing out that the transcriptomic PI3K score in this prior study correlated with the corresponding RPPA measurements of pathway activity, which in turn was positively associated with INPP4B and PTEN loss, and to some extent PIK3CA amplification. All three events are frequently observed in TNBCs, in particular, and support the notion that signalling activity “dose” should be considered as a separate factor in breast cancer etiology and, possibly, treatment choices. Adding to this, a recent study also suggested increased mutant PIK3CA mRNA expression in ER- breast cancer samples, in both METABRIC and TCGA cohorts (Correia et al. 2021 bioRxiv doi: 10.1101/2021.03.05.434137). This could further contribute to the increased PI3K pathway score observed in this tumour subset.*

2) Can the predicted activity score of the PI3K pathway be of any help in the clinical decision making? As compared to an exome capture to assess the mutational status of the PIK3CA gene (E542K, E545K, H1047R residues...) for example. The prediction from the authors' findings is that ER- patient would benefit from PIK3CA inhibitor as they have the highest activity for this pathway. Is there any evidence from preclinical or clinical studies to support such prediction? the authors should at least discuss this point.

**AUTHORS’ RESPONSE:**

*It is commonly assumed that cancer-associated activation of a signalling pathway predicts sensitivity to its pharmacological inhibitors. However, we have previously observed apparent rewiring of the transcriptome in homozygous PIK3CA^H1047R^ iPSCs, which is no longer reversible on PI3K inhibition despite being triggered by its initial activation* [8]*.*

*In their pan-cancer analyses, Zhang et al.* [6] *also noted a negative correlation between the CMAP-based transcriptomic PI3K score and PI3K pathway inhibitor sensitivity in cultured cells. In this study, transcriptomic and proteomic scores in snapshot cell-based measurements are moreover not necessarily correlated; this is not surprising, since the former integrates pathway activity over time whereas the latter reflects acute signalling state, as outlined in the Introduction of the current manuscript. Sensitivity to a pathway inhibitor may also differ according to the phenotypes that are used as evaluation criteria. For example, it is possible to suppress tumour growth temporarily, as is often observed with PI3K pathway inhibitors in a clinical cancer setting, but without elimination of stem cell-like cancer cells, tumour regrowth and metastasis are almost invariable.*

*Given these observations we prefer not to speculate that tumours with high PI3K scores (e.g. TNBC, HER2+) will necessarily be more sensitive to PI3K pathway inhibition, as the converse could be true. The data could also be used to raise the question whether PI3K inhibitors should be combined with pro-differentiation therapy in tumours with high PI3K activity and stemness scores, such as TNBCs where PI3K pathway inhibitors have had modest or no success* [9–12]*. While this is beyond the scope of our current study, we note that work from the laboratory of Ramon Parsons in mouse models of metastatic breast cancer has demonstrated the potential utility of combining PI3K and BET inhibitors to overcome a MYC-dependent feedback mechanism that limits the benefit of single-agent PI3K pathway inhibition in this context* [13]*. We have included this additional information in the revised Discussion.*

*While our main aim with the current study was to obtain definitive evidence for a quantitative link between PI3K signalling strength and stemness in breast cancer, we nevertheless hope that the findings may spur evaluation of the potential clinical benefits of integrating transcriptomic indices of PI3K pathway activation with conventional genetic information. More generally, the diagnostic and therapeutic benefits of combined, comprehensive genomic and non-genomic analyses were recently demonstrated in a prognostically unfavourable rare cancer patient population* [14]*, and are likely to apply more broadly. We have added this additional information to the revised Discussion.*

3) The authors should treat their human iPSCs with heterozygous or homozygous PIK3CA H1047R mutations with different concentrations of alpelisib, to find out if the PI3K activity score correlates with dependence to the PI3K pathway?

**AUTHORS’ RESPONSE:**

*Due to practical limitations imposed by the current pandemic, we have unfortunately been unable to access the iPSC facilities where such work could be performed. We are moreover unsure that this experiment will add much value to the current manuscript given that the iPSCs in question are unlike cancer cells and thus do not tolerate high doses of alpelisib. Even relatively low doses of the drug can be cytotoxic to these cells (Madsen R.R. 2018 Doctoral Dissertation https://doi.org/10.17863/CAM.35662). It will thus be more valuable to investigate the Reviewer’s suggestion in breast cancer-relevant models, however these have yet to be engineered with allele dose-dependent PIK3CA^H1047R^ expression and are thus beyond the scope of the current manuscript.*

4) Figure 5 revealed correlation of the Plurinet signature with cell proliferation and metabolism which is already know from the literature, this should be at least mentioned in the text.

**AUTHORS’ RESPONSE:**

*This information has now been added to the last Results section.*

5) Dosage relationship has been previously shown for PTEN loss and stemness in mouse models. The author should mention these studies.

**AUTHORS’ RESPONSE:**

*We have now included this information in the revised Introduction.*

Minor points:

- Figure 1D and E: Wouldn't it be more accurate to replace the legend PI3K_mSigDb by PI3K_GSVA? Because PI3K_Progeny refers to the model used to calculate much like GSVA?

**AUTHORS’ RESPONSE:**

*Since we use GSVA with multiple different signatures, we think replacing “mSigDb” with “GSVA” for the hallmark PI3K signature only may be equally confusing. We have now replaced “mSigDb” with “Hallmark”, to better reflect the source, and have additionally specified in the figure legend that the PROGENy PI3K signature and OCLR stemness scores were calculated using alternative methods to GSVA as reflected in their names.*

- Why is the correlation value different from Figure 1 Panel A (0,49) to Figure 1 panel D (0.5)?

**AUTHORS’ RESPONSE:**

*This appears to be due to a rounding that is intrinsic to the calculations performed within the code that generates the correlation plots in Fig. D & E. It is unclear why the correlation map code would perform this rounding for the Rho value between the PI3K_Hallmark and Stemness_PLURINET signature scores in METABRIC, yet keep the exact original value when repeating the analysis with TCGA data. Nevertheless, the difference is minor and consistent with the 0.49 value so we think it does not warrant additional troubleshooting.*

- Line 187-190: Please specify in the text which PIK3CA mutations you are analyzing, same for line 200

**AUTHORS’ RESPONSE:**

*This has now been specified for lines 187-190. We believe it has been specified sufficiently for line 200, especially with the additional information provided in the Methods section and in the accompanying analysis code.*

- Figure 2 A, C, D: Please, provide percentages. For example, Line 165 - 167 : results are described in percentage while the figure is showing absolute values, please specify % in the figure as well for the sake of clarity

**AUTHORS’ RESPONSE:**

*This has now been done for Fig.A and C. For D, adding the percentages would result in too much clutter, so we decided to keep it as is.*

- Figure 4 B: Please, be consistent, by either writing ns (non-significant) in all ns comparisons or writing the p-value in all ns comparisons.

**AUTHORS’ RESPONSE:**

*This has now been addressed.*

- Figure 3: The statistical calculations for the median comparisons are between what groups? Low versus high, Low versus intermediate or both?

**AUTHORS’ RESPONSE:**

*The log-rank test was performed by the survdiff() function [R survival package] and results in a global p-value whether to accept or reject the null hypothesis that the three different groups come from the same distribution. It is not a pairwise comparison and is therefore not presented as such in the figure.*

- Sentence starting in line 246 and ending in line 248 is speculative and should be moved to the discussion.

**AUTHORS’ RESPONSE:**

*This has now been done.*

- Figure 4 : stats are missing in panel C and lower panel A.

**AUTHORS’ RESPONSE:**

*This has now been addressed.*

**Reviewer #3**

*Evidence, reproducibility and clarity:*

See below

*Significance:*

I think this is a very useful and interesting manuscript. Using a single "PI3K-AKT-mTOR signaling" gene signature, the authors analyzed both TCGA breast and METABRIC breast cancer cohorts. They concluded that tumors with "single" PIK3CA hotspot mutations have a lower "PI3K score", compared to ones with either wildtype or "multiple" PIK3CA hotspot mutations. In doing this, the authors have very forcefully captured the idea that single copy PK3CA mutations alone are not likely to be extremely useful in characterizing tumor response to therapy. This idea is quite apparent in both the preclinical and clinical literature. Thus preclinically it has long been apparent that knock in of a single allele of a tumor mutant of PIK3CA as was done by the Parsons lab is a relatively benign perturbation resulting in mice that get tumors with low penetrance and long latency while inducible transgenes or PTEN loss give tumors of short latency and high penetrance. Recent clinical/preclinical findings cited by the authors suggest a similar idea, but the concept is nicely documented here, using clinical and preclinical data, along with the extremely important idea that this signature could be used to design treatment. However there are significant areas for improvement:

1. As the authors note, different types of breast tumors give markedly different data in this analysis. Shouldn't breast cancer be treated like the separate diseases that it is, instead of lumping all together. It is well known that breast tumors are a mixture of apparently different types of disease, from the perspectives of gene expression, genetics, and treatment in the clinic. For example, triple-negative breast cancer, which scores very highly in the analysis presented in this MS, is more similar to high-grade serous ovarian tumors and squamous lung carcinoma in terms of gene expression, rather than to ER+ breast cancer (TCGA, Nature 2012; Hoadley, Cell 2014, 2018) These tumors also have the poorest prognosis compared to either HER2+ or ER+ breast cancer. Triple negative tumors also frequently feature loss of PTEN which has a strong 'PI3K" signature ( again see Parsons lab data) but is hard to follow from genetic data in breast cancer, since loss of PTEN expression is not genetically driven. With such an overwhelming (epi-)genetic heterogeneity, there is no point of analyzing gene set enrichment in the total population of breast cancer. Instead, the authors would be well advised to concentrate on the ER+ subtype, which is the subtype with the most frequent PIK3CA mutations and is also the only type of malignancy where a p110 alpha inhibitor is currently approved.

**AUTHORS’ RESPONSE:**

*Several of these important points are now addressed in our response to point 2 from Reviewer 2, and the manuscript has been updated accordingly.*

*We also agree that the non-genetic heterogeneity associated with PTEN inactivation poses a formidable challenge when it comes to predicting PI3K pathway activity on the basis of genomic data alone. In fact, this is an excellent point that to some extent supports one of our key messages, namely that genomic classification of tumours with respect to PI3K pathway components should be supplemented with functional indices of pathway activity (ideally over time, which is where transcriptomic readouts may be particularly useful as outlined in lines 99-112 in the manuscript). We have attempted to emphasise this further in the revised Discussion.*

*We do, however, retain the view that looking at all breast cancer subtypes is important for the current manuscript since our main aim was to determine if there is evidence for a systematic relationship between indices of PI3K signalling strength and stemness directly in human tumours. Towards this aim, we also show subtype-specific analyses. In fact, the data from TNBCs are perhaps the strongest evidence for a strong correlation between the PI3K/AKT/mTOR score and stemness. This information would mostly have been lost if only analysing genetic data, both with respect to PIK3CA mutations and PTEN loss (which often occurs non-genetically as the Reviewer rightly points out). Given that TNBCs have very poor prognosis and lack effective treatment strategies, we believe that our analyses in this context are worthwhile presenting as they should prompt further investigation of the PI3K signalling-stemness link in this context, including potential preclinical studies of combining PI3K inhibitors with pro-differentiation/epigenetic therapies (e.g. BET inhibitors, see also our response to Reviewer 2, point 2).*

2. The authors used a single "PI3K-AKT-mTOR" signature to derive PI3K activity score, but they also mentioned that "Notably, the separate mTORC1 gene signature exhibited a much stronger correlation 245 (Spearman's rho = 0.7) with the stemness score compared with the PI3K_AKT_mTOR signaling signature used to derive the PI3K activity score". The authors should explain how the choice of a "PI3K-AKT-mTOR signaling" signature is rationalized, and how this signature was profiled in the cited Cell System paper. It would be much more satisfying to see the data analyzed using multiple PI3K signatures. The authors could present both the results with signatures that bolster their argument and those that do not-allowing them to compare and contrast the usefulness of the signatures. And to be fair, they need to replace "PI3K activity" in the title and elsewhere with "PI3K-AKT-mTOR activity" if they chose to stick with the use of this particular signature.

**AUTHORS’ RESPONSE:**

*We have addressed this point in our response to point 1 from Reviewer 2, including the use of two additional PI3K signature which bolster our conclusions. Regarding the choice of the PI3K_AKT_mTOR signature over the mTORC1 signature, the former was chosen because we are interested in capturing PI3K pathway-related effects as broadly as possible, whereas an mTORC1-only signature would restrict us to one particular downstream effector (which is also activated downstream of diverse nutritional inputs). Given the limitations of computational analyses, we can only speculate at this stage that the higher correlation between the mTORC1 signature score and the stemness score may reflect that the mTORC1 is the main component underlying the mechanistic link between PI3K pathway activation and stemness.*

*We have also made it more explicit that we are looking at PI3K/AKT/mTOR signalling, including a change to the title. We hope this is sufficiently clear now, but are happy to amend further if required.*

A smaller point: It is not clear from the manuscript how the authors determined if a given TCGA/METABRIC tumor sample has single or multiple copies of PIK3CA mutations, or if it is possible to infer "mutant PIK3CA allele dosage" from the two datasets. If a tumor is annotated for single PIK3CA hotspot mutation (as analyzed in Figure 4A, B), how do we know it is not a homozygous mutation? Likewise, for tumors with an annotation for 2 or 3 PIK3CA mutations, how do we know if mutations happen on both alleles of the same tumor cells rather than in different cancer cells that individually have only one mutation. Importantly, for tumors with single mutations, the allele frequency could be vastly different, a factor that should be taken into consideration as well. The authors need to present their work in discussing these issues.

**AUTHORS’ RESPONSE:**

*We thank the Reviewer for this very important point, which we have also attempted to address in the revised Methods section. We obtained allele gain/amplification information for METABRIC tumours from cBioPortal and for TCGA from our previous analyses (Madsen et al. 2019 PNAS* [15]*). In both cases, such information relies on a well-established computational deconvolution method known ASCAT (allele-specific copy number analysis of tumours), which seeks to assign accurate allelic copy number to individual genomic regions while also estimating and adjusting for both tumour ploidy and normal cell admixture (Van Loo et al. 2010 PNAS* [16]*). Importantly, we have considered genome doubling as a potential confounding factor and found no evidence for it (Fig. 4B). For breast cancer data from TCGA, we also removed all low-purity samples from the analyses (described in Methods). With respect to allele dose for each individual mutation, this information is provided by the ASCAT analyses and is taken into account in the presented analyses. In other words, tumours with multiple copies of the same hotspot mutations are categorised as such and are not grouped together with the single-mutation tumours.*

*It remains difficult to ascertain that two or more mutations are present in the same cell as opposed to different cells. We note, however, that we have focused exclusively on hotspot PIK3CA variants for ‘allele dosage’ analyses, and such variants have been estimated to be clonal in breast cancer (McGranahan et al. 2015 Science Translational Medicine* [17]*). Importantly, McGranahan et al. also estimated that of the 225 non-silent PIK3CA mutations (hotspot and non-hotspot) in their TCGA ER+ breast cancer dataset, only 19 (8.4%) were subclonal, with the number falling to 1/27 (3.7%) for ER- tumours (see Fig. 4A in McGranahan et al.* [17]*). This strengthens the notion that our analyses are unlikely to be confounded by tumour mosaicism for different PIK3CA mutations.*

*Given our selective focus on hotspot mutations, these are most likely in trans according to prior work based on additional phasing information* [18–20]*.*

**References**

1	Sanchez-Vega, F. et al. (2018) Oncogenic Signaling Pathways in The Cancer Genome Atlas. Cell **173**, 321-337.e10.

2	Malta, T. M. et al. (2018) Machine Learning Identifies Stemness Features Associated with Oncogenic Dedifferentiation. Cell **173**, 338-354.e15.

3	Liberzon, A. et al. (2015) The Molecular Signatures Database Hallmark Gene Set Collection. Cell Syst. **1**, 417–425.

4	Schubert, M. et al. (2018) Perturbation-response genes reveal signaling footprints in cancer gene expression. Nat. Commun. **9**, 20.

5	Jin, X. et al. (2020) A metastasis map of human cancer cell lines. Nature **588**, 331–336.

6	Zhang, Y. et al. (2017) A Pan-Cancer Proteogenomic Atlas of PI3K/AKT/mTOR Pathway Alterations. Cancer Cell **31**, 820-832.e3.

7	Koboldt, D. C. et al. (2012) Comprehensive molecular portraits of human breast tumours. Nature **490**, 61–70.

8	Madsen, R. R. et al. (2021) NODAL/TGFβ signalling mediates the self-sustained stemness induced by PIK3CA H1047R homozygosity in pluripotent stem cells. Dis. Model. Mech. dmm.048298.

9	Kim, S. B. et al. (2017) Ipatasertib plus paclitaxel versus placebo plus paclitaxel as first-line therapy for metastatic triple-negative breast cancer (LOTUS): a multicentre, randomised, double-blind, placebo-controlled, phase 2 trial. Lancet Oncol. **18**, 1360–1372.

10	Oliveira, M. et al. (2019) FAIRLANE, a double-blind placebo-controlled randomized phase II trial of neoadjuvant ipatasertib plus paclitaxel for early triple-negative breast cancer. Ann. Oncol. **30**, 1289–1297.

11	Garrido-Castro, A. C. et al. (2020) Phase 2 study of buparlisib (BKM120), a pan-class I PI3K inhibitor, in patients with metastatic triple-negative breast cancer. Breast Cancer Res. **22**, 1–13.

12	Schmid, P. et al. (2020) Capivasertib plus paclitaxel versus placebo plus paclitaxel as first-line therapy for metastatic triple-negative breast cancer: The PAKT trial. J. Clin. Oncol. **38**, 423–433.

13	Stratikopoulos, E. E. et al. (2015) Kinase and BET Inhibitors Together Clamp Inhibition of PI3K Signaling and Overcome Resistance to Therapy. Cancer Cell **27**, 837–851.

14	Horak, P. et al. (2021) Comprehensive Genomic and Transcriptomic Analysis for Guiding Therapeutic Decisions in Patients with Rare Cancers. Cancer Discov. candisc.0126.2021.

15	Madsen, R. R. et al. (2019) Oncogenic PIK3CA promotes cellular stemness in an allele dose-dependent manner. Proc. Natl. Acad. Sci. **116**, 8380–8389.

16	Van Loo, P. et al. (2010) Allele-specific copy number analysis of tumors. Proc. Natl. Acad. Sci. **107**, 16910–16915.

17	McGranahan, N. et al. (2015) Clonal status of actionable driver events and the timing of mutational processes in cancer evolution. Sci. Transl. Med. **7**, 1–12.

18	Vasan, N. et al. (2019) Double PIK3CA mutations in cis increase oncogenicity and sensitivity to PI3Kα inhibitors. Science (80-. ). **366**, 714–723.

19	Saito, Y. et al. (2020) Landscape and function of multiple mutations within individual oncogenes. Nature **582**, 95–99.

20	Gorelick, A. N. et al. (2020) Phase and context shape the function of composite oncogenic mutations. Nature.
