# Supplementary material for: Positive correlation between transcriptomic stemness and PI3K/AKT/mTOR signaling scores in breast cancer, and a counterintuitive relationship with PIK3CA genotype
Source: PLoS Genet. 2021 Nov 11;17(11):e1009876. doi: 10.1371/journal.pgen.1009876 (PMC8584750; doi:10.1371/journal.pgen.1009876)
Supplement: Attachment — Submitted filename: 2021-09_Rebuttal_PI3K_stemness_comp_MS_RRM_v2.docx [file pgen.1009876.s006.docx]

------------------------------------------------------------------------------

**Attn:**

Peter McKinnon, PhD
Section Editor: Cancer Genetics
PLOS Genetics

David Kwiatkowski, PhD
Section Editor: Cancer Genetics
PLOS Genetics

Dear Dr. McKinnon and Dr. Kwiatkowski,

We are grateful for your consideration of our manuscript and for the opportunity to submit a revised version addressing the concerns of Reviewer 1. To do this, we instigated a collaboration with Prof Alex Toker and his group, enabling us to add the much-needed experimental evidence that our PI3K signalling score has biological significance. We provide further details about this revision in the following response to Reviewer 1.

Other changes include a minor change to the title, typo corrections and change from British to American English.

We believe that this revision has strengthened the manuscript substantially and hope that you will now consider it acceptable.

Sincerely yours,

Ralitsa R. Madsen

(*on behalf of all authors)*

**Reviewer #1**

*The revised manuscript from Madsen et al does honestly quite little to address what I still believe to be reasonable concerns about the paper. I do agree with the other two reviewers that the conceptual point, which I understand to be that higher dimensional markers of PI3K activity (“scores”) are likely to be more clinically useful and indicative of response to anti-PI3K drugs, in comparison to the simple single genetic mutation marker, is a useful and important concept. This paper does have analyses that support this idea. Yet, I still contend that an important control is to ask the question of how many other such “scores” show the types of trends that are observed, but by chance? This is not simply looking for another alternative PI3K score as was done by the authors. Perhaps, this kind of rigor is beyond scope in the opinions of the editors and other reviewers, but to me it does seem warranted to understand the fidelity of the finding, particularly given the absence of experimental follow up. Further, I apologize for the lack of clarity in the type of experimental evidence I thought might be needed to make the paper more sufficiently convincing.* ***To me, cell line and cell culture experiments would be perfectly adequate…I was not stating new animal / tissue / human data was needed for this paper. However, I still find a major gap in the paper to be that no phenotypic or signaling markers of PI3K/mTOR/AKT activity (e.g. S473 phosphorylation on AKT) are measured to show the scores have validated meaning, and/or whether cell lines with different mutations in PI3K and scores have differential responses to PI3K inhibitors. Those should be relatively straightforward cell culture experiments.*** *The only cell line data I found in the paper was for stem cell lines that were homo or heterozygous for PI3K H1047R (Fig. 4D), showing they have different PI3K and stemness scores. Thus, while overall it seems that I am in agreement more than not with the other two reviewers, I may still differ in my opinions as to additional analyses that would help make this paper convincing enough to have wide impact. Yet, I am OK if the others disagree with that assessment and find the revised manuscript to be satisfactory.*

**AUTHORS’ RESPONSE:**

*We thank the Reviewer for this clarification.*

*We have now addressed the request highlighted in bold by including experimental data from breast epithelial MCF10A cells with genetic PI3Kα activation. Specifically, we used the cells developed by Lien et al.* [1]*, featuring retroviral overexpression of PIK3CA^H1047R^*, *alongside the corresponding empty vector (EV) controls. We chose to study these cells in growth factor-replete (more physiologically relevant) conditions, well aware that this would minimise differences in PI3K/AKT pathway activation; if such differences are nevertheless picked up by our method for inferring PI3K pathway activation from transcriptomic data, it would attest to the sensitivity of the approach. We performed three independent experiments in which the effect of PIK3CA^H1047R^ overexpression was evaluated in the presence of DMSO or the p110α-specific inhibitor BYL719, with sample collection after 48 h and 120 h of treatment. The resulting data are included in a new Figure 2. In addition to biochemical characterisation of PI3K pathway activation, the cells were also processed for RNA sequencing, with the t values from subsequent differential gene expression processing used for gene set enrichment analysis (GSEA) with the “HALLMARK_PI3K_AKT_MTOR_SIGNALING” gene signature as done for the breast tumour samples (note that these were processed with GSVA given that GSEA only allows for predefined experimental comparisons, whereas GSVA builds on the GSEA principle but extends to single samples provided n>30). We also included a comparison with the “HALLMARK_MTORC1_SIGNALING” signature.*

*In short, we demonstrate that the “HALLMARK_PI3K_AKT_MTOR_SIGNALING” gene signature can be used as a transcriptional footprint of PI3K pathway activation: it correctly predicts activation of the pathway in DMSO-treated PIK3CA^H1047R^ MCF10A cells relative to EV controls; conversely, it also correctly predicts pathway inhibition upon p110α inhibition with BYL719. In contrast to the “HALLMARK_MTORC1_SIGNALING” signature, results with the “HALLMARK_PI3K_AKT_MTOR_SIGNALING” gene signature were also robust to differences in gene filtering criteria – these data can be found in a revised Supplementary Figure S1.*

*We have also updated the Methods section with information related to these substantial revisions (cell culture, Western blotting, RNA sequencing), including additional technical explanations that seek to clarify the distinction between GSVA and GSEA.*

*Finally, to ensure adequate interpretation of our findings, we have also made minor changes to the Discussion, emphasising “the need for future benchmark studies that establish the relationship between the absolute magnitude of individual transcriptomic scores for the PI3K pathway and corresponding biochemical activity, and how this relationship may be affected by crosstalk with other pathways that converge on similar transcriptional outputs.”*

**References**

1	Lien, E. C. et al. (2017) Oncogenic PI3K promotes methionine dependency in breast cancer cells through the cystine-glutamate antiporter xCT. Sci. Signal. **10**.
